# Explaining the prevalence of marital conflict: conceptual bifurcation and sociological explanations

**DOI:** 10.3389/fsoc.2025.1490385

**Published:** 2025-01-31

**Authors:** Wing-Chung Ho

**Affiliations:** City University of Hong Kong, Kowloon, Hong Kong SAR, China

**Keywords:** marriage, marital conflict, conceptual bifurcation, contextual explanation, evolutionary explanation

## Abstract

Sociologists have investigated extensively marital conflict which is supposedly “antithesis” of marriage. However, there is little systematic reflection on how the coexistence of universal marriage *and* prevalent spousal discord in diverse cultural settings can possibly explained sociologically. This conceptual paper aims to address this issue by first critically reviewing how scholars have assessed the prevalence of marital conflict in human societies. This review is then extended to the conceptual elusiveness in gauging “marital conflict,” arguing that the concept has been inadvertently bifurcated as (i) a *constituent* (oft-represented as a single global continuous measure) of certain critical consequential events within a marriage (e.g., divorce); and (ii) a *predisposition* (oft-represented in terms of a set of multifarious binary variables) in pair-bonding relationships that increases the likelihood of the occurrence of certain critical consequential events. Such conceptual bifurcation sheds light on two board distinctive approaches—roughly termed contextual and evolutionary—through which the coexistence of marriage formation and martial conflict can be sociologically explained. Implications are briefly discussed.

## Introduction

The institution of marriage, usually considered one of the key foundations of social structure, has long been a focal point of modern sociological theories (Durkheim, 1921; Murdock, 1949; Marx and Engel, 1848; Bourdieu, 1996). Marriage refers to the legally recognized bond between two or more persons, which includes a range of culturally accepted rights and obligations (Leach, 1955). Researchers, however, generally do not bother to make guesses into the *raison d'etre* of the universality of conjugal relationship; rather, they presume that this is the fact out there, and they only need to make good guesses into the structures and processes behind the emergence of different marriage forms, the complex interactive processes within marriage, and the impacts marriages have on the actors and societies. This presumption possesses truism in reality. The study by Walker et al. (2011, p. 1) on present-day hunter-gatherers around the world suggests marriage is “a fundamental cornerstone of human economic, social, and kinship networks”, which infers a deep evolutionary history of institutionalized pair-bonding that stems back at least to early modern humans (see also, Chapais, 2008, 2010). Such relatively long-term cooperative partnership—be it monogamous, polygynous, or polyandrous—between the two sexes is often viewed as a human universal (Marlowe, 2000; Murdock, 1949; Scelza, 2022). In fact, getting married is still almost a life mission for every contemporary human as over 80 percent of people marry at least once by age 40 (Willoughby et al., 2015, p. 189). Despite general declines in marriage rates since the end of WWII, an overwhelming majority of individuals are expected to marry or eventually engage in other forms of partnership, such as cohabitation (Thornton and Young-DeMarco, 2001).

Logically, the sociological exploration of marriage is incomplete without addressing supposedly its “antithesis”—marital conflict. The situation is analogous to Durkheim's investigation of suicide against the prevalence of social solidarity. According to the *Oxford Leaner's Dictionaries*, “prevailing” means “existing or most common at a particular time.”^1^ One of the goals of this conceptual paper is to argue that marital conflict is prevalent among all marriages, which carries similar academic interest to the study of suicide in relation to social solidarity, although suicide is considered less widespread within societies. In fact, sociological literature has been replete with discussions on explaining marital discord. Drawing upon the role theory of Parsonsian functionalism, many studies, for example, examine the relations between wives' income and marital conflict (see Winslow, 2011 for a review). Seeing earnings as a potential source of marital conflict, some sociologists further cast this model in a feminist light, considering income difference between spouse as a significant source of power and gender inequality in society (e.g., Rogers and DeBoer, 2001). Other studies further find that marital conflict is associated with men's leisure time usage (Collis, 1999), the division of labor between household and paid work (Kluwer et al., 1996), differences in earning power and its changes between spouses (Winslow, 2011), cross-border marriages (Choi and Cheung, 2017), and migration (Umubyeyi, 2019). The constructionists, on the other hand, deem the roles within a couple as the outcome of continuous interactive (re-)negotiation (Potuchek, 1992; Zvonkovic et al., 1996). Constructionists thus deem marital conflict as depending on the extent to which spouses achieve a consensus on their roles (Kluwer et al., 1996). Major (1993), adopting this logic, even suggests that wives can be relatively satisfied with an uneven distribution of unpaid family labor, as long as it aligns with their socialization and relationship expectations (see also, Thompson, 1991).

The abovementioned sociological theorizing offers sufficient explanatory power if marital conflict is sporadic or even exceptional among human conjugal relationships, akin to the relationship between suicide and social solidarity in the case of Durkheim. But what if marital conflict is commonplace phenomenon in human societies? In other words, how can sociologists possibly explain the rather paradoxical coexistence of both marriage formation and marital conflict in diverse cultural settings? In the literature, no systematic reflection has been made into this issue, and this paper aims to fill this gap. To proceed further, this paper first review how scholars assess the prevalence of marital conflict in societies. This review is then extended to the elusiveness of the term “marital conflict,” and, as will be made apparent, pinpoints that the concept has been inadvertently *bifurcated*. What it means is that, on the one hand, marital conflict is considered a *constituent* of certain critical consequential events (CCEs). CCEs, here, refer to the incidents triggered by specific actions taken by one or both partners that exert a negative, nature-changing impact on a marital relationship, such as divorce, infidelity, or intimate partner violence. Being conceptualized in such a way, marital conflict is often represented in terms of a single global continuous measure with marital “conflict” and “harmony” located at the two opposite ends of the spectrum. On the other hand, marital conflict is conceptualized as a human *predisposition* which makes possible the occurrence of certain CCEs in pair-bonding relationships. In this light, marital conflict is often represented in terms of a set of multifarious binary variables through which an actor can possibly consider his/her marriage both “conflictual” and “harmonious” at the same time. This paper suggests that such conceptual bifurcation sheds light on two board distinctive approaches—roughly called context and evolutionary—that the coexistence of marriage formation and martial conflict can be explained sociologically.

## The prevalence of marital conflict

In this section, I will draw from the existing literature to assess the prevalence of marital conflict through two approaches: (i) primarily quantitative, statistical accounts of the occurrence of CCEs, and (ii) qualitative, descriptive, or historical accounts of the norms and institutions that directly or indirectly facilitate CCEs. The first approach mainly utilizes data from Western, industrialized societies, while the second focuses on non-WEIRD (non-Western, educated, industrialized, rich, and democratic) societies. This dual approach highlights universal patterns and cross-cultural differences essential for understanding variation.

Taking the first approach into account, I view that the sociology-related literature summarizes broadly five ways—two positive and three negative—to gauge or proxy the prevalence of “marital conflict”: marital happiness, marital satisfaction, divorce, infidelity, and intimate partner violence; these are worthy of examining one by one.

Speaking of marital happiness, the national surveys of the United States are by far the most comprehensive database on the subject in human history. The question being asked is: “Taking things all together, how would you describe your marriage? Would you say that your marriage is very happy, pretty happy, or not too happy?” Statistics over the past four decades reflect a highly consistent result. On average, around 63 percent of people report being “very happy” in their marriage, which is already considered high. If this figure is combined with those indicating “pretty happy,” the percentage averages over 96 percent, and this overwhelming trend has persisted for the past 40 years.^2^ This finding inevitably represents strong counter-evidence against the prevalence claim about marital conflict. However, many scholars quickly realize that demographic data collected in the same period indicate that about one in two marriages ends in divorce (e.g., Glick, 1984; Raley and Bumpass, 2003; Cherlin, 2009). Some studies even suggest an even higher divorce rate with two-thirds of all new marriages ending in dissolution (e.g., Martin and Bumpass, 1989). The findings indicating that almost all couples reporting being “happily married”, *and* half of them ending up divorce suggest that those who report being “very happy” or “pretty happy” about their marriage in surveys are not so. Surprisingly, no systematic effort has been made to determine the exact number of divorces involving statistically “happy couples”. In his study on how parental discord and divorce affect children's wellbeing, Amato's (2002) data accidentally revealed the variability in marital discord prior to divorce among 1,130 couples during the 1980s and 1990s. Pursuing the samples over 12–17 years after the first interview in 1980, Amato found that 264 of the couples in question got divorced (23.4%). My reworking of Amato's data indicates that among the 264 divorced couples, 82.2 percent rated their marriage as “very happy” or “pretty happy” 1 month to 5 years prior to divorce. This means that over 80 percent of couples who were unhappy enough to get divorced had deemed themselves “happy” at one point.^3^ In fact, respondents' consistent over-positive interpretation of their marital happiness has prompted scholars to consider—rather counterintuitively—“pretty happy” as equivalent to “not too happy” in their analyses by coding them into the same statistic category (e.g., Whitton et al., 2013, p. 279). The figures and insights derived from the above discussions lead me to estimate, with reasonable confidence, that *at least half* of the respondents in national surveys of the United States are *actually unhappy* with their marriages.

Speaking of another indicator of marital conflict “marital satisfaction,” Karney and Bradbury (2020, p. 101) have observed that the decline of marital satisfaction over time is nearly “as close to a truism as exists in marital research” regardless of whether the marriage is new or old (see also, Jackson et al., 2014). Some studies report a curvilinear relationship between marital satisfaction and the stage in the life cycle, with a substantial decline during childbearing and childrearing phases, followed by a leveling off during the launching stage, and then substantial increases through pre-retirement and retirement stages (Stattin and Klackenberg, 1992; Weisfeld and Weisfeld, 2002). VanLaningham et al. (2001), however, disagree with the U-shape view of marital satisfaction. Their study based on data from a national 17-year, 5-wave panel sample suggest that a significant negative effect of marital duration on marital satisfaction remains after other key life-course variables are controlled. Lupri and Frideres (1981, p. 284) also pinpoint that the U-shape curve is likely a consequence of methodological bias of elective survival. It means that divorced/separated individuals and dissatisfied couples, or those with domineering spouses, are more prone to disappear from the samples of long-term marriages.

How about divorce as an indicator of marital conflict? As hinted before, in the United States, 39–50 percent of first marriages end in divorce, with the divorce rate increasing to 60 to 70 percent for second marriages and reaching as high as 70–73 percent for third marriages (Amato and James, 2010; Schoen and Canudas-Romo, 2006; Putnam, 2011; Luscombe, 2018). The estimated average length of marriage ranges from 6.8 to 8 years (Kitson et al., 1985; McDevitt, 2013). The divorce rate in the United States has improved in recent years as baby boomers, who tended to marry young, began to die (Pelley, 2018). Similar trends have been observed in the United Kingdom, with the divorce rate rising during the second half of the twentieth century and beginning to drop in the 2010s (Wood, 2018; Luscombe, 2018). Marrying later in life is apparently a strong protective factor for lasting marriage, with scholars estimating the “sweet spot” for a lasting marriage to be between the ages of 28 and 31 (Marsee, 2018). However, the declining divorce rate in the United States in the late 2010s, from 50 percent decades ago to the range of 25 to 39 percent, does not necessarily indicate that marital relationships have become more harmonious. In fact, there is actually less unhappy marriages to break up in modern societies, where more and more people choose not to get married or cohabit with an unmarried partner. But, unfortunately, cohabitation is not a protective factor for sustained marriage, as living together prior to marriage is found to increase the chance of divorce by 40 percent (McDevitt, 2013).

Next, infidelity. A comprehensive study by Previti and Amato (2004) conclude that the relationship between deteriorating marital satisfaction and extramarital sex is a classic “chicken or egg” question. They find that infidelity is *both* a cause *and* a consequence of relationship deterioration based on their 17-year longitudinal study covering 1,475 couples in the United States from the 1970s to the 1990s. Fisher (2016) argues that adultery is a prevalent tendency in human cultures, with underestimated statistics in the United States indicating that 20 to 40 percent of men and 20 to 25 percent of women have had an extramarital affair in heterosexual marriages. She expresses surprise at findings from scientists reporting that 56 percent of men and 34 percent of women in their study were unfaithful to their partners, yet they were self-reportedly in “long-term *happy* partnerships” (Fisher, 2015, p. 53; original emphasis; see Glass and Wright, 1985). In a lengthy literature analysis, Fisher (2016, p. 80–81) summarizes nearly 20 factors that have been significantly linked to extramarital affairs, including unfulfilled needs, lack of love, insufficient sex life, boredom, poor communication, low perceived ability to live happily, desire for self-expansion, openness to new experiences, being less conscientious, less agreeable, and more neurotic, imbalanced social power between spouses, perceiving oneself as more socially desirable than one's spouse, alcoholism, depression, narcissism, chronic illness of a spouse, the frigid personality of the wife, and constant travel by a spouse. She then concludes that adultery occurs in every culture around the world as mate poaching is found to be common in thirty cultures other than that of the United States. From Fisher's analysis, it is clear that any married person can find themselves falling into one or more of the adultery-prone categories at some point in their lives, which somehow confirms the general tendency of humans to engage in sexual behavior outside of marriage. In fact, if the definition of adultery for a married person includes “romantic infidelity,” such as romantic exchanges without sexual intercourse, an even higher proportion, if not virtually *all*, of married men and women can be labeled as unfaithful at certain points (Tsapelas et al., 2010; Fisher, 2011).

Finally, regarding the conflict form of intimate partner violence, similar to the case of divorce, no study has been specifically designed to measure its prevalence among self-reported “happy” couples. However, several studies do suggest that a considerable proportion of victims of domestic violence tend to remain in their marriages and even consider their relationships as “happy.” For instance, studies have found that battered wives in cultures with strong collectivism tend to stay with their abusive partners to avoid the negative impact of divorce on their families (Abdul Ghani et al., 2015; March, 2018, p. 37). In a study by Vázquez et al. (2015, p. 24) that involved 136 low-income women who were victims of intimate partner violence in Nicaragua, 36.4 percent of them reported being “quite a lot” or “a lot” satisfied with their marital relationships when asked. My past fieldwork experiences in Hong Kong suggest that nearly half of battered wives considered their marriages “not so bad” because their husbands would do something positive, such as buying them a gift after the physical assault (Ho, 2012).

Turning to the second approach that concerns non-WEIRD societies, scholars have reached a consensus regarding the practice of polygyny, which is permitted in over 70 percent of societies with a significant majority (80%−90%) of men engage in monogamous marriage, primarily due to insufficient resources to support multiple wives simultaneously (Murdock and White, 1969; Frayser, 1985; Flinn and Low, 1986; Van den Berghe, 1979; Binford, 2001; Marlowe and Berbesque, 2012; Kenrick et al., 2013; Kramer et al., 2017; Scelza, 2022). Most of these cultures exhibit serial monogamy, where individuals—whether men or women—engage in monogamous relationships at different times throughout their lives, potentially bearing offspring with multiple partners. The norms that support this practice include the acceptance of divorce (remarriage) and extramarital sex. In her analysis of 186 societies within the Standard Cross-Cultural Sample, Betzig (1989) found that only five societies considered divorce to be uncommon or nonexistent. Among the various factors contributing to divorce, infidelity emerges as a predominant cause (Shackelford, 1998). Although fidelity is generally regarded as a fundamental principle of marriage, 39 percent of 185 cultures were reported to be accepting of extramarital sex (Ford and Beach, 1951). Furthermore, another cross-cultural study indicated that extramarital sex was rare or absent for men in only 20 percent of societies, while for women, this figure was 27 percent (Broude and Greene, 1976). Evidence of widespread acceptance of divorce and extramarital relationships thus suggests a likely prevalence of marital conflict. This conflict likely underpins the social norms that facilitate divorce and extramarital sex, indicating that these practices are not exceptions but integral aspects of marital dynamics in different cultural settings.

It is important to recognize that the widespread presence of norms and institutions addressing marital conflict does not necessarily imply the prevalence of CCEs, such as divorce, infidelity, and intimate partner violence. Scholars have suggested that these occurrences are mediated by various demographic and ecological factors, including adult sex ratios and resource distribution (Scelza, 2022, p. 533), as well as cultural influences, such as Christianity's characterization of extramarital sexual relationships as sinful, which emphasizes chastity and fidelity within marriage (Pedersen, 2014). But, cross-cultural demographic evidence indicates that when married couples are “given the chance”—such as in contexts where the sex ratio is more balanced, resources are more abundant, or cultural attitudes lean toward gender equality—marital conflict in terms of divorce and infidelity tends to increase (Blurton Jones et al., 2000; Schacht and Kramer, 2016; Yodanis, 2005). Therefore, the quantitative data from Western industrialized societies, along with widespread norms and institutions surrounding divorce (remarriage) and extramarital relationships in non-WIERD societies, suggest both direct evidence and indirect cultural affordances that highlight how culture provides individuals with specific opportunities and constraints shaping their behaviors and interactions related to marital conflict. This understanding forms the basis for my argument regarding the prevalence of marital conflict across different cultures in this paper.

## The elusiveness of “marital conflict”

Then, how can the coexistence of prevalent marital conflict and marriage be explained sociologically? To answer this question, one needs to take a step back to clarify how the concept of “marital conflict” has been operationally defined and empirically measured. In previous studies, marital conflict is generally defined as “[emotional] distress [over some specific matters] results from couples' aversive and ineffectual response to conflict” (Fincham and Beach, 1999, p. 48; see also, Koerner and Jacobson, 1994, p. 208). Marital conflict essentially refers to the experience of negative emotion by one or both spouses in a martial relationship which is *causally related*, in one way or another, to the occurrence of certain CCEs, such as divorce, infidelity or violence. The conceptual juxtaposition between marital conflict and the occurrence of CCE is crucial because if marital conflict does not lead to certain negative, relationship-transformational consequences, it should not be termed as “conflict”. For example, if most marital conflicts had been transformed into opportunities for cooperative interaction between the two actors (e.g., Kelley and Thibaut, 1978); they should have been termed “tiffs” or “small spats” and the discussions of these *happy quarrels* between lovers should have been given a lot less sociological attention than they have actually had. This view echoes how Amato understands spousal commitment. He argues that the concept should be understood in terms of its *consequences* for relationships. He astutely states: “This is why the military hands out medals after the battle and not before. In a similar sense, we cannot tell whether spouses are committed to their marriages until they are put to the test” (Amato and Hohmann-Marriott, 2007, p. 308).

One should note that previous studies usually consider marital conflict and its causal relation with the occurrence of CCE—either explicitly or implicitly—in terms of two board heuristic models: deterministic and mechanismic. Deterministic models presume marital conflict to be a *constituent* predictive of certain CCEs through a series of linear causal links. It is often represented as a single global continuous measure in determining the occurrence of certain CCEs. The respondents are, for instance, asked to rate a single statement in a Likert scale of to what extent the they are satisfied or happy with their marriage akin to the National Survey question on marital happiness. Or, similar to the Kansas Marital Satisfaction (KMS) Scale, respondents are asked to rate three relationship satisfaction questions: (i) How satisfied are you with your marriage/relationship?; (ii) How satisfied are you with your marriage/relationship with your wife/husband/partner?; and (iii) How satisfied are you with your with your wife/husband/partner as a spouse/partner? (Schumm et al., 1986). The ratings are then computed into a single KMS score which are usually found significantly correlated to the occurrence of CCE such as divorce (e.g., Schumm et al., 2000).

Another common deterministic approach of marital conflict is the ENRICH Marital Satisfaction (EMS) Scale (Fowers et al., 1996). It is a 15-item scale that includes the Marital Satisfaction and Idealistic Distortion scales from the marital inventory ENRICH (Fowers and Olson, 1989). The ENRICH Inventory is composed of 125 items which cover various dimensions of marital life, including communication, conflict resolution, financial management, sexual satisfaction, parenting, religious beliefs, and overall relationship satisfaction. The Marital Satisfaction scale is a 10-item Likert format measure of global marital satisfaction. The Idealistic Distortion scale is a 5-item measure used as a correction for the tendency to over-report marital satisfaction. The EMS scale thus results in a single continuous measure of marital conflict that is subjectable to mathematical summation and subtraction; and then to statistical correlations with—and external validation by—the occurrences of separation and divorce.

One popular usage of the ENRICH Inventory is to transform the scores of the 125 items (10 dimensions) in into a five-category typology of couples. These categories, according to Olson and Fowers (1993) consist of: (i) “vitalized” couples who report high relationship quality on all ENRICH dimensions; (ii) “harmonious” couples who have relatively high relationship quality across all dimensions; (iii) “traditional” couples who have scores that are slightly above average with markedly higher scores on parenting and religion scales; (iv) “conflicted” couples who are characterized by moderately low scores on all but the equalitarian roles scale; (v) “devitalized” couples who have the lowest scores on every ENRICH dimension. These five categories are then put in a—once again—linear continuous order (or an ordinal order, to be exact) with “vitalized” couples considered the most harmonious relationship down to the “devitalized” couples at the opposite end. Subsequent statistical analyses indicate positive significant correlations between each category and the occurrences of separation and divorce in different cultural settings (e.g., Olson and Fowers, 1993; Cohen et al., 2010).

The abovementioned measures of martial conflict all render any marital relationship as a *single* point (or category) in a linear spectrum with “conflict” and “harmony” located at the opposite ends. Evidently, such an approach is merited in many ways, especially, in conducting various hypothetico-deductive analyses to identify key contributing factors of marital conflict, and by association the occurrence of CCE. For instance, compared to other marriage categories, statistical correlations show that individuals in both “conflicted” and “devitalized” couples tend to have been married for a shorter period of time, have a shorter acquaintance prior to marriage, and a higher incidence of racial and religious heterogamy (Olson and Fowers, 1993, p. 204; see also, Rogers and Amato, 1997; Choi and Marks, 2011).

Some scholars, however, cast doubts over the deterministic view of marital conflict as they see in the real world the two incidences—the occurrence of marital conflict and its related CCEs—are casually related *not* in an “if A, then B” manner. Rather, whether the negative emotion of one or both spouses (as the start condition) will eventually bring about certain relationship transformation events (as the finish condition) involves a *mechanism* that entails complex interactions of multifarious factors not even known to the actor or the researcher. Space constraint forbids me to elaborate the nature of mechanism and its attendant mechanismic explanation from scratch (see reviews in Hedström and Ylikoski, 2010; Knight and Reed, 2019). Simply put, a mechanismic explanation, according to Nicholson (2012, p. 159; see also, Machamer and Darden, 2000, p. 3), is *not* deterministic as to exhaust “all the causal relations necessary for the production of” a particular phenomenon; but merely to “individuate and causally relate the entities and activities that are responsible for” that phenomenon.

Along the mechanismic view, the causal relation between marital conflict (an emotion) and the occurrence of a CCE (an event triggered by specific actions) is more of *probabilistic* rather than deterministic in nature. Marital conflict is thus *not* considered as a necessary or sufficient cause for the occurrence of a CCE; rather, it *predispose*s individuals with certain qualities to interact with a multitude of other factors which in turn enhance the occurrence of certain CCEs. A closer look at the literature, one will find that the conceptual presumption of such the mechanismic view on marital conflict is not uncommon. For example, as mentioned before, one revealing statistic indicates that on average about half of individuals engaging in infidelity rate their marriage as “happy” (Glass and Wright, 1985). Besides, the stark statistical incongruity between so many “happy” marriages ending in divorce has raised the eyebrows of and remains a puzzle to researchers. Amato (2002), for example, also observes that many couples can divorce without explicit preceding signs of marital distress. Scholars have also yet to reconcile the seemingly oxymoron of having couples reporting higher spousal satisfaction in remarriages (Buunk and Mutsaers, 1999; Jose and Alfons, 2007, p. 77) *and* having a higher risk for divorce than those in first marriages (Bramlett and Mosher, 2001; Bulanda and Brown, 2007; Whitton et al., 2013). On the other hand, certain couples experience regular conflicts manage to maintain rather fulfilling and/or long-lasting marriages (Hetherington and Kelly, 2002). One of the reasons, as proposed by Kitson et al. (1985, p. 258), is that there may be a time-lag between marital conflict and divorce known as “emotional divorce”; they observe that “[t]he majority of the divorced are able to document when their marriages started to sour, often many years before the decisions to divorce” (see also, Fulton, 1979; Kelly, 1982). Some studies, as mentioned earlier, even find that battered wives tend to remain in their marriages and stay with their abusive partners to avoid the negative impact of divorce on their families (e.g., Abdul Ghani et al., 2015). These studies, together with some others, have identified a number of reasons which explain why so many unhappy marriages remain intact, including the number of dependent children (Betzig, 1989), resource consideration (Vázquez et al., 2015), and social norms (Ho, 2012).

Contrary to the deterministic view, the mechanismic view of marital conflict is operationalized in terms of a set of multifarious binary variables; rather than a single global measure. This distinction can be easily grasped with the following illustration. In line with the ENRICH inventory, scholars have long distilled four key components of marital conflict, namely: (1) communication (e.g., does my partner make me laugh often?); (2) housework allocation (e.g., is my partner willing to spend time with the kids?); (3) resource sufficiency (e.g., does my partner make enough effort to ensure the family has enough money to spend now and in the future?); and (4) sexuality (e.g., do my partner and I have good sex in terms of quality and quantity?) (e.g., Yelsma, 1984). Individuals in couples are free to rate their relationship along these four conflict components in positive or negative terms. A deterministic view of marital conflict will average out the pluses and minuses an individual's ratings of these components and come up with a single marital conflict score. A mechanismic view, however, will conceive the score in each component as conceptually distinct; for example, a wife may rate her relationship highly positively because her husband gives her a lot of money to spend, but at the same time she rates negatively for her husband in the other three components. Therefore, unlike the deterministic view of marital conflict, an actor can possibly consider his/her marriage both “good” and “bad” at the same time. Moreover, a mechanismic researcher does not bother to calculate a composite conflict score for the actor, or pigeonhole him/her into one single conflict category. It is because whether the emergence of martial conflict will actually lead to the occurrence of certain CCEs is probabilistic subjected to the complex interactions and processes involving multifarious factors at the personal, interpersonal, and societal levels in relation to a specific actor; rather than determined by a statistically deduced Pearson's *r*.

That being said, the mechanismic view of marital conflict is inevitably criticized for lacking statistical robustness in conducting statistical inference analyses that presume linear causality between conflict-related independent variables and the occurrence of CCE. However, it is better-positioned to offer support for understanding the conundrum of why so many couples who have had minimal overt conflict yet ultimately divorce; or, as put Amato (2007, p. 306) it, the “sudden jumps” or “nonlinear discontinuous changes in marriage.” It, for example, echoes the catastrophic theory on close relationships which suggests that marital satisfaction can decline drastically due to sudden, significant negative events; or accumulated unresolved conflicts within the relationship (Rusbult et al., 1991; Braithwaite et al., 2016). The mechanismic perspective also provides one with more conceptual clues to come to grips with the still mythic “transformative processes in marriage” through which a spousal relationship abruptly turns sour (Fincham et al., 2007, p. 275).

The conceptual bifurcation of marital conflict thus far illustrates two distinctive definitions of the term, resulting in two different ways of gauging its prevalence as a phenomenon. The deterministic view defines marital conflict as a constituent, a correlate of the occurrence of CCE. Whether marital conflict and its associated CCEs (e.g., divorce) are ubiquitous in society is basically an empirical question subjected to contextual analyses pertinent to a specific time and place; and the empirical answers are always presented in terms of a matter of degree (i.e., higher or lower). The mechanismic view, on the other hand, considers marital conflict in an idiosyncratic, situational, and transient manner. It refers to the fleeting moments of dissatisfaction felt by one or both spouses in a specific situation that leads to the ideation on the part the actor to alter the relationship nature, which in turn increase the likelihood of—rather than determines—the actual occurrence of CCE. Being so defined, marital conflict is by nature an integral aspect of marital dynamics as it is akin to what Fincham (2003, p. 23) claims: “Marital conflicts can be about virtually anything,” and they inevitably predispose the actors toward relationship change.

## The bifurcated explanations

The conceptual bifurcation of marital conflict allows us to broadly categorize the current sociological explanations of prevailing marital conflict against the universality of marriage into two camps. The first can be generally termed “contextual explanations” which attend to the changing social ideology and/or structure, such as the rise of individualism and gender inequality. The second can be labeled “evolutionary explanations” which views the ubiquity of marital conflict as potentially offering benefits to human population in terms of enhancing survival and reproduction.

### Contextual explanations

Comparing statistics documented across decades, sociologists attribute the rise of marital dissatisfaction and divorce to the rise of “expressive individualism” since the WWII (e.g., Giddens, 1992), and many studies even suggest that the phenomenon is particularly that of the culture of the United States, which, according to Amato (2010, p. 1455), “differs in fundamental ways from marriage in other Western countries” (see also, Cherlin, 2009; Whitehead, 1998). They acknowledge the rise of individualism has raised the standards for an acceptable spouse and marriage. This shift is juxtaposed with the belief that individuals have the right, and perhaps even the obligation, to pursue personal happiness and psychological growth. Many individuals now thus seek a partner who will aid in personal development, facilitate personal fulfillment, and contribute to the realization of one's full potential as a human being. The ideal spouse is now valued for being a soulmate, rather than mainly for resource security or emotional comfort. As a result, a significant number of Americans choose to divorce each year due to a perceived lack of fulfillment in their marriages. Statistics have shown that the United States stands out as a country where partnerships are formed, dissolved, and reformed at a higher frequency than in most other nations.

From a historical perspective, sociologists have noted how ideals of mating and male-female relationships have evolved over time: antiquity's worship of heroic prowess in love gradually gave way to medieval notions of courtly and companionate affection, subsequently transforming again into modern ethos of romantic and unanimous rapport (Bandlien, 2005; Boase, 1977; Giddens, 1992; Singer, 1984). Underpinning these shifting paradigms have been transformations in the intimate realm: we have progressed from the highly structured connubial bonds within traditional society, through the moderately formatted matrimonial arrangements of eighteenth and nineteenth century sentimental love (which nevertheless retained various presumptions regarding the naturalness of gender roles), toward today's ideal of “pure relationship” or “confluent love,” where increasingly more such constructs become negotiable between autonomous companions. Consequently, “motherhood,” “the family,” “masculinity,” and “femininity,” all previously considered inherent conditions or types, are now being treated by some as lifestyle selections (Larsen, 2023). Consequently, modern formal marriages have increasingly become what Finkel (2019) famously postulated: “all-or-nothing” suggesting partners anticipate acquiring all their self-actualization needs satisfied by their mate and perceive divorce as an unhesitant option if those needs go unfulfilled, rather than compromising or working through troubles as in times past.

### Evolutionary explanations

Biological evolutionists posit that the purpose of getting married is to maximize the reproductive fitness for both parents by recruiting two individuals who can aid in childrearing and maintain a committed alliance between them (Kleimen, 1977; Tsapelas et al., 2010, p. 175). While the universality of marriage is so explained, the conflict between the spouses is *also* predisposed in the heterosexual conjugal relationships. According to Buss, marital conflict is originated in the fundamental differential sexual strategies. Women desire greater emotional commitment and resource investment, while men prioritize short-term sexual needs. These differences then lead to post-marriage conflicts over sex, finances, division of labor, and childcare (Buss, 2016; see also, DeLecce, 2018). Such differences lay down the foundation that “the dissolution of long-term mating relationships … [becomes] universal across cultures” (Buss, 2016, p. 269).

Then, what distinctive survival and reproductive benefits does prevailing marital conflict, in terms of infidelity or divorce, bring about? Thus far, evolutionary scientist Fisher has made a remarkable thesis along this line. She first observes the widespread and persistent phenomenon of infidelity—in the form of “clandestine adultery”—in monogamous avian and mammalian species, including humans. She then argues that *both* sexual infidelity *and* pair-bonding constitute the dual or mixed reproductive tactics that explain the modern worldwide conjugal dissolution peak after 3–4 years of marriage (Fisher, 2016, p. 144). Fisher's dual reproductive strategy has been confirmed by multiple scholars, including Chapais (2013), Kramer and Russell (2015, p. 78), and Petrella (2005, p. 174).

The evolutionary perspective thus considers marital conflict as a human predisposition which is compatible to the prevalence of serial monogamy—a sociocultural formation that potentially brings about higher evolutionary advantages than other institutional arrangements to the human species. Humans are thus inclined “to fall in love, form a pair bond, leave this relationship after 3–4 years (often after bearing a single child), and then fall in love anew and bond again” (Fisher, 2016, p.135). And, marital conflict is logically human's predisposition; otherwise, in the case of lifelong monogamy, good genes are likely to be confined to a single biparental—happy and faithful—marital relationship with limited number and genetic variety of offspring. Genetic variety, in turn, effectively increases the potential for survival against bacteria, viruses, and other parasites that are biologically harmful to humans (Fisher, 2016, p. 53; Miller, 2000, p. 186–187). Notably, this idea to consider the formation of sociocultural instructions as a strategy to enhance human evolutionary fitness echoes the very recent sub-discipline of sociology known as evolutionary sociology (e.g., Turner and Machalek, 2018; Schutt and Turner, 2019, p. 372; Ho, 2023).

## Conclusions

This paper presents evidence suggesting that marital conflict is prevalent across diverse cultural settings, advocating for a deeper, more systematic exploration of its coexistence within marriages. In this conceptual analysis, the elusiveness of “marital conflict” is unpacked, revealing how it has inadvertently been bifurcated into two distinct ways to conceptualize and evaluate (measure) marital conflict: first, as a constituent of certain critical consequential events (CCEs) within marriage, such as divorce; and second, as a predisposition in pair-bonding relationships that increases the likelihood of these CCEs occurring.

This way of conceptualizing and evaluating marital conflict also presents a meaningful analogy for understanding the evaluation of health. Just as marital conflict can be conceptualized through bifurcated lenses, health has been debated as being perceived differently by diverse stakeholders, among whom clinical professionals and individual patients are two pivotal interlocutors (Canguilhem, 1991, 2012). To measure health, therefore, similar to the conceptualization of marital conflict discussed herein, fundamentally depends on the epistemological questions: “What is health?” and “How is health constructed?” (Turchi et al., 2022, p. 3). Both marital conflict and health thus represent a kind of conceptual reality situated in a dimension where intellectual processual dynamics generate meaning. Such phenomena are not merely naturalist entities but constructs shaped by gnoseological categories and linguistic practices from different perspectives.

In sum, by delineating different perspectives along this line, this paper highlights the nuanced nature of marital conflict, which may have been previously overlooked by sociologists and other relationship scientists. The proposed bifurcated explanations—contextual and evolutionary—illustrate that while evolutionary dispositions may drive the formation of *both* marital bonds *and* conflict, contextual predictors, including cultural and ideological influences, shape the institutions and norms that govern the expression of these dual practices. It therefore remains challenging to clearly identify the root causes of marital conflict within the universal institution of marriage.

## Footnotes

^1^See Oxford Learner's Dictionaries: https://www.oxfordlearnersdictionaries.com/definition/english/prevailing?q=prevailing.

^2^Data source: https://gssdataexplorer.norc.org/trends/Gender%20&%20Marriage?measure=hapmar.

^3^Although Amato's data were collected two to three decades ago, it remains relevant today. The key evidence is that his dataset exhibits striking similarities with national surveys in terms of the samples' proportions that described their marriage as “very happy” (62.1%) or “very happy or pretty happy” (95.2%) before divorce.
